# Arousal Regulation by the External Globus Pallidus: A New Node for the Mesocircuit Hypothesis

**DOI:** 10.3390/brainsci13010146

**Published:** 2023-01-14

**Authors:** Zhong Sheng Zheng, Nicco Reggente, Martin M. Monti

**Affiliations:** 1 Research Institute, Casa Colina Hospitals and Centers for Healthcare, Pomona, CA 91767, USA; 2 Institute for Advanced Consciousness Studies, Santa Monica, CA 90403, USA; 3 Department of Psychology, University of California Los Angeles, Los Angeles, CA 90095, USA

**Keywords:** arousal, GABAergic neurons, globus pallidus externa, mesocircuit hypothesis, pallido-cortical pathway, sleep, thalamic reticular nucleus, zolpidem

## Abstract

In the decade since its debut, the Mesocircuit Hypothesis (MH) has provided researchers a scaffolding for interpreting their findings by associating subcortical-cortical dysfunction with the loss and recovery of consciousness following severe brain injury. Here, we leverage new findings from human and rodent lesions, as well as chemo/optogenetic, tractography, and stimulation studies to propose the external segment of the globus pallidus (GPe) as an additional node in the MH, in hopes of increasing its explanatory power. Specifically, we discuss the anatomical and molecular mechanisms involving the GPe in sleep-wake control and propose a plausible mechanistic model explaining how the GPe can modulate cortical activity through its direct connections with the prefrontal cortex and thalamic reticular nucleus to initiate and maintain sleep. The inclusion of the GPe in the arousal circuitry has implications for understanding a range of phenomena, such as the effects of the adenosine (A2A) and dopamine (D2) receptors on sleep-wake cycles, the paradoxical effects of zolpidem in disorders of consciousness, and sleep disturbances in conditions such as Parkinson’s Disease.

## 1. Introduction

Subcortical-cortical interactions have long been appreciated for their crucial role in supporting arousal, an important aspect of consciousness. The functional integrity of these large-scale interactions has accounted for gradations of consciousness in both healthy and patient populations [1,2,3,4]. Indeed, the Mesocircuit Hypothesis (MH), a dominant framework for understanding the loss and recovery of consciousness in the context of severe brain injury, focuses on the role of subcortical-cortical recurrent interactions [5], placing particular emphasis on the central thalamus, whose diffuse cortical projections [6] are susceptible to injury-induced deafferentation. According to MH, such damage, particularly on top of the widespread damages typically seen in severe brain injury, can result in decreased excitatory output from the thalamus to both the cortex and striatum. This reduced output further decreases the activity of the striatal medium spiny neurons (MSNs). These neurons, when activated, would normally inhibit the internal segment of the globus pallidus (GPi), which in turn disinhibits the central thalamus. However, without sufficient input to the MSNs, the GPi is not inhibited and leads to excessive inhibition on the thalamus and, subsequently, a global metabolic depression that is proportional to the depth of the impairment of consciousness [5,7]. While appealing and well-grounded in empirical support, the MH, as is the case in all models, is an incomplete simplification. Here, we address the MH’s oversimplified consideration of the basal ganglia (BG) structures. While aligning with the traditional motor view of the BG nuclei, the MH has yet to incorporate the novel and rapidly growing literature that highlights a role for the external segment of the globus pallidus (GPe) in arousal.

The human globus pallidus (GP) (In rodent literature, the GPe is referred to as globus pallidus and the GPi as entopeduncular nucleus, whereas in human literature, the globus pallidus represents the amalgamation of the external (GPe) and internal (GPi) pallidal subdivisions. In this review, we will adhere to the primate/human nomenclature), also known as the pallidum, is a crucial constituent of the BG circuitry and can be anatomically, neurochemically, and functionally divided into external (GPe) and internal (GPi) segments. The GPe has by and large been positioned as a relay structure in the so-called indirect pathway loop uniting the cortex, basal ganglia, and thalamus, and primarily supporting motor suppression. However, there is mounting evidence that the GPe supports a wider repertoire of functions. Specifically, recent work suggests a previously unappreciated role of the GPe in the maintenance of electrocortical and behavioral arousal [8,9,10,11,12]. Additionally, empirical findings suggest the existence of direct (i.e., not mediated by the GPi/substantia nigra pars reticulata) GPe connections with the cortex [13,14,15] and thalamus [15,16,17,18], as well as their involvement in modulating arousal and sleep [8,12].

In what follows, we begin by reviewing the evidence for the connection between the GPe and arousal. We then examine the relationship between the GPe and disorders of consciousness (DOC), considering both existing research and the potential benefits of incorporating the GPe into the MH with regard to its explanatory power.

## 2. GPe in Arousal/Sleep-Wake Regulation

The BG circuitry can be viewed as a template function for transition control, regulating excitatory and inhibitory inputs [19]. This framing helps to highlight the BG’s classic role in initiating and supporting the state shifts between rest and movement, and also positions it as an interface for the loss and recovery of consciousness, such as during sleep and wakefulness, as well as a range of other cognitive and limbic functions [20]. Indeed, recent animal studies have unveiled several BG structures to be crucial for sleep-wake regulation, in particular the dorsal (caudate, putamen) and ventral striatum and the GPe [8,9,10,12].

Investigation into the GPe’s role in arousal must also consider the striatum, particularly its dorsal segment (dSTR), given their linked circuitry and the striatum’s purported involvement in sleep-wake control. The dSTR predominantly contains GABAergic medium spiny neurons that project to the GPi (direct pathway) and GPe (indirect pathway) [21]. Neurotoxic lesions in the dSTR lead to a reduction and destabilization of wakefulness, with rodents exhibiting 15% less wakefulness, a fragmentation of sleep-wake behavior, and a slowing of EEG rhythms towards the delta band [8].

Neurotoxic lesions to the GPe causes both a promotion and destabilization of wakefulness, with rodents exhibiting a 46% increase in wakefulness, frequent sleep-wake transitions, and a decrease in NREM sleep bout duration [8]. On the other hand, the direct optogenetic stimulation [10] and deep brain stimulation [9] of rodent GPe neurons have resulted in increased sleep [9,10] and EEG delta power [10]. Interestingly, neurotoxic lesions to regions intimately connected to the GPe and striatum (i.e., GPi, subthalamic nucleus, substania nigra reticulata) did not significantly alter the sleep-wake cycles [8]. Consistent with these animal findings, a recent case report found that the deep brain stimulation (DBS) of the GPe improved insomnia symptoms in a patient with Parkinson’s Disease (PD) [22].

These lesion and stimulation findings may be serving as exogenous probes of the endogenous chemical mechanisms that support the transitions between sleep and wakefulness. Adenosine has long been implicated in sleep control, with bodily levels increasing as a function of continuous periods of wakefulness, accompanying the sensation of sleepiness [23]. A_2A_ receptors (A_2A_R), a subtype of adenosine receptors, play a major role in sleep induction and are densely expressed in the striatum [24], primarily in the striato-pallidal pathway involving the GPe [12,25]. Yuan and colleagues [12] found that the chemogenetic excitation of A_2A_Rs in the rostral and central subregions of the dSTR produced an increase in NREM sleep, whereas inhibiting those subregions decreased NREM—a finding that did not extend to the caudal striatum. In the same study, the research team found the A_2A_R neurons in the rostral dSTR to preferentially form inhibitory synapses with parvalbumin positive (PV+) neurons in the rostral GPe, whereas the A_2A_R neurons in the caudal dSTR formed inhibitory synapses on the PV- neurons in the caudal GPe. Accordingly, the direct inhibition of the PV+ neurons in the GPe mimicked the effect of rostral and central striatal A_2A_R neuron excitation through increased sleep. Moreover, the lesioning of the PV+ neurons in the GPe abolished the typical increase in NREM sleep seen when activating the striatal A_2A_R neurons [12]. These insights position the PV+ neurons in rostral GPe as a necessary component in the sleep-wake circuitry and permit the sleep-wake functionalities associated with A_2A_R in the rostral dSTR to be extended to the GPe.

Dopamine (DA) also plays a key role in sleep-wake behavior [26]. The loss of DA neurons in the substrantia nigra compacta (SNc), which densely innervate the dSTR, is the main pathological characteristic of PD, where up to 90% of patients report sleep disturbances [27]. Considering that the GPe’s neuronal activity is modulated by DA from the SNc via the D_2_ receptors in the striato-pallidal neurons [28,29], as well as the GPe’s purported involvement in sleep-wake control, it stands to reason that manipulating the SNc DA inputs to the striato-pallidal pathway could affect sleep-wake outcomes. Indeed, the optogenetic stimulation of SNc’s dopamine terminals in the rat dSTR increased total sleep in the proceeding 24 h by 69%, accompanied by increased EEG delta power (0.5−3 Hz) [10]. Additionally, in the same study, the authors selectively ablated SNc DA neurons and their afferents to the dSTR, which not only caused sleep-wake fragmentation and disrupted diurnal sleep-wake alternation, but also resulted in significant sleep reduction. The number of remaining SNc DA neurons were found to negatively correlate with an increase in wakefulness [10]. These findings led the authors to suggest that nigrostriatal DA acting on GPe may help to promote sleep. 

The D_2_ and A_2A_ receptors both play a role in promoting sleep—a shared implication that could be interactive due to the A_2A_-D_2_ heteromers in the striatum [30], with an emphasis on the dendritic spines of the striato-pallidal neurons [31]. Indeed, dopaminergic signaling reduces as a function of adenosine binding [32]. Given the relay of communication from the SNc to the dSTR to the GPe, there is a plausible role of the D_2_ and A_2A_ receptors in the sleep disturbances seen in PD patients with SNc deterioration. The D_2_ and A_2A_ receptors are increased in the dSTR of PD patients [33], and it has been suggested that overactive striatal A_2A_ receptors, as is the case in PD, may lead to the increased suppression of the GPe and contribute to the common effect of excessive daytime sleepiness seen in PD [34,35]. 

While the literature lacks a site-specific convergence at consistent levels of resolution to draw conclusions regarding these mechanisms, one can confidently assert that the A_2A_ and D_2_ receptors, the dSTR-GPe pathway (anterior/rostral subregion), and the GPe PV+ neurons are critical variables in the equation. In the next section, we discuss the BG circuitry, particularly the efferent paths of the GPe that may directly impact cortical activity, and how its design may allow for a homeostatic harmony that initiates and perpetuates sleep-wake cycles.

## 3. Proposed Striato-Pallidal Circuitries for Promoting Sleep

It has been proposed that recurrent BG-cortical interactions may influence cortical and behavioral arousal [11]. According to the classic direct/indirect BG models, BG outputs to the cortex are primarily mediated by projections from the GPi/SNr to the thalamus, to facilitate or suppress motor activity, respectively. However, it remains unlikely that this conventional BG output pathway is responsible for regulating cognitive arousal, given that: (i) lesions of GPi/SNr do not significantly affect sleep-wake behavior; and (ii) the GPi’s connections appear to be more restricted to motor-related structures [15,36], consistent with the GPi/SNr’s classic role in motor control. The indirect motor suppressing BG pathway involving the GPe, (i.e., GPe—subthalamic nucleus—GPi/SNr) could potentially serve to inhibit motor activity during sleep. 

On the other hand, BG outputs to the cortex can also be mediated by the GPe, which has direct projections to the frontal cortex and indirect outputs to the cortex via the thalamus. Additionally, there appears to be a population of prototypical GPe neurons that receive cortical inputs and target both the cortex and thalamic reticular nucleus (TRN) [37], providing a circuit that supports both the reciprocal cortico-pallido-cortical interactions and the mediation of thalamic sensory gating, which is necessary for maintaining sleep [38].

### 3.1. Cortico-Striato-Pallido-Cortical Route

Direct GPe projections to the cortex were previously thought to be a part of the basal forebrain’s (BF) cortical projections [39]; however, a recent tracing study verified that pallido-cortical GABAergic neurons receiving striatal input were well within the boundaries of the GPe and appear to be distinct from the cholinergic BF neurons near the ventral and medial borders of the GPe [13]. While a subset of cholinergic GPe neurons [14] exists, the rostral part of the GPe, which contains the PV+ neurons involved in sleep regulation [12], are predominantly GABAergic [13]. Where the BF’s cortical projections are diffuse, the GPe’s GABAergic cortical projections, which receive inputs from the dSTR, are more localized to the frontal cortex [11,13,14].

**Figure 1:**
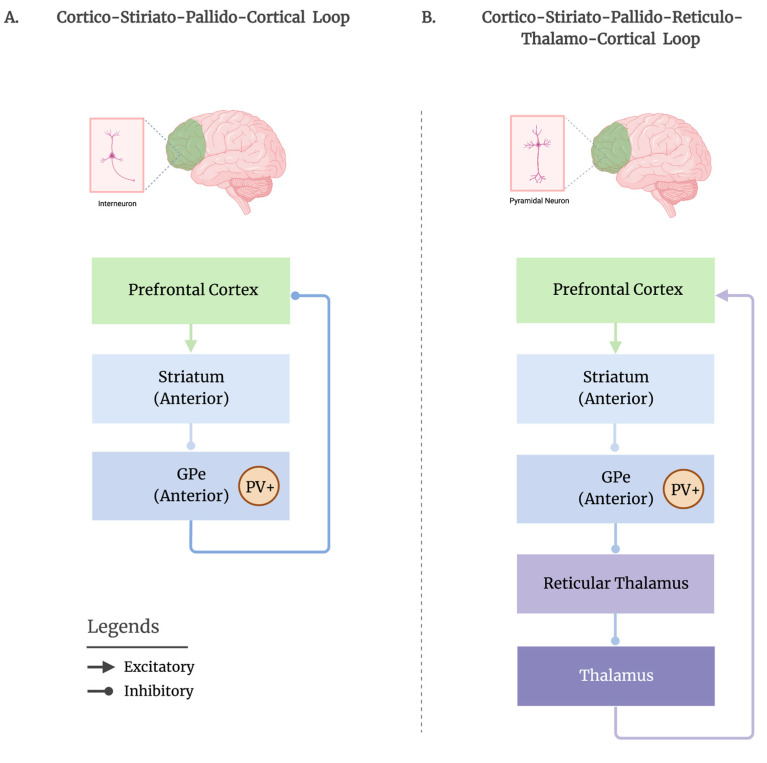
Two potential routes by which the GPe may mediate cortical activity are proposed. (**A**). Following an anterior (rostral) topographical organization, the frontal cortex sends excitatory projections to the anterior portion of the dorsal striatum (caudate, putamen), which then sends inhibitory projections to the anterior GPe, where the PV+ neurons reside. The inhibition of the GPe may lead to the disinhibition of the frontal GABAergic interneurons, permitting them to suppress the firing of pyramidal cells to promote sleep, via the direct pallido-cortical route. (**B**). Another route by which the GPe may influence sleep could be through its connections with the thalamic reticular nucleus (TRN) (i.e., reticular thalamus). Following the striatal inhibition of the GPe, the GPe releases its inhibition on the TRN. Then, the TRN is free to suppress (i.e., gate) the thalamo-cortical neurons and prevent the excitation of pyramidal cells in the cortex to disrupt sleep. Figure is adapted from “Basal Ganglia Motor and Non-motor Loops”, by BioRender.com, accessed on 29 December 2022. Retrieved from https://app.biorender.com/biorender-templates, accessed on 29 December 2022.

The exact mechanism of how the GPe regulates sleep-wake activity remains unclear. One hypothesis is that the inhibition of the GPe neurons by the dSTR may lead to the disinhibition of the GABAergic interneurons in the frontal cortex, resulting in the suppression of the pyramidal cells and the promotion of sleep (Figure 1A). Recent studies have shown that the direct GABAergic pallido-cortical neurons target both the GABAergic interneurons and, to a lesser extent, the glutamatergic pyramidal neurons, which are found to be reciprocally connected—an excitatory-inhibitory connectivity profile that could help stabilize electrocortical states during sleep by regulating circuit-wide activity to sub-arousal thresholds [13,14,38]. Moreover, direct pallido-cortical projections have been identified to be denser in the deep cortical layers [8,13], in particular layer V, the lamina being crucial for generating EEG waves [40] and a primary candidate for facilitating brain-wide synchrony, such as that seen during sleep [41]. The direct projection from the GPe to the frontal cortex, bypassing the thalamus, may serve as a rapid route for suppressing cognition to initiate the sleep process. As such, during sleep, in humans, slow-wave activity (SWA) appears first and predominates in the frontal brain areas and decreases along an anterior-posterior gradient [42,43]. This propagation of SWA in the posterior brain regions might reflect the subsequent quiescence of sensorimotor activity during sleep. 

### 3.2. Cortico-Striato-Pallido-Reticulo-Thalamo-Cortical Route

Another route by which the GPe may regulate cortical activity and facilitate sleep could be through its direct output to the TRN—a thin layer of GABAergic neurons surrounding and innervating the dorsal thalamus that controls the thalamocortical information flow [44]. Direct connections between GPe and TRN have been observed in primates, cats, and rodents [16,17,18,45]. As the GPe exerts a GABAergic influence on the TRN, electrical and chemical perturbations of the GPe can directly influence the spontaneous firing rates of the TRN neurons; as a result, GPe excitation decreases, whereas this inhibition increases the TRN activity [46,47], suggesting the GPe’s tight control over the TRN’s activity. The TRN has been consistently recognized for its role in generating sleep spindles, delta sleep rhythms and slow oscillations [48,49]. The direct activation of the TRN neurons has resulted in increases in NREM sleep [50,51]. More recently, the TRN has also been implicated in producing local variations in sleep rhythms [52,53], a phenomenon known as local sleep, which challenges the established notion that sleep is an exclusively global state. An important mechanism involved in ensuring sleep continuity and preventing minor sensory stimuli from reaching the cortex and disrupting sleep is through sensory gating, which is a crucial role of the thalamus [54]. Thalamic sensory gating is believed to be regulated by the prefrontal cortex through the basal ganglia, via the dSTR—GPe—TRN pathway [55]. Although the precise mechanism remains unclear, we speculate that following the inhibition of the GPe by the dSTR, the TRN becomes disinhibited and is free to suppress (gate) the thalamocortical outputs, thus preventing the excitation of the cortical pyramidal cells from disrupting sleep (Figure 1B). 

## 4. Globus Pallidus in Consciousness Disruption

In parallel with the growing evidence supporting the notion that the GPe is a crucial component of arousal regulation, a number of recent studies also suggest a role of the GP in subserving aspects of consciousness and its disorders. In healthy volunteers, pallido-cortical functional connectivity has been associated with the loss and recovery of consciousness during anesthesia [56]. In patients with DOC, both behavioral arousal [57] and the perturbational complexity index (PCI) [58], a neural measure capable of reliably discriminating between conscious and unconscious patients, have been shown to correlate with the degree of atrophy in the GP and dorsal striatum. Although the GPe and GPi were not separated in these studies, the atlas-based coordinates from the latter study [58] indicated that over 70% of the atrophy was within the GPe. Additionally, in an EEG study on chronic DOC patients, pallidal atrophy was associated with decreasing the total density across both the power spectrum and the beta-to-delta (i.e., fast-to-slow) ratio [59], a metric previously implicated in behavioral arousal [8,57]. 

Zolpidem (commonly known as Ambien)—a GABA-A agonist commonly used to treat insomnia—has paradoxically promoted arousal in some DOC patients [60] and has been associated with inappropriate motor activation, such as that seen in somnambulism (sleep walking; [61]). Using the MH, it has been suggested that zolpidem can serve as a substitute for striatal inhibition on the GPi, given the abundance of GABA-A receptors in the GP [62], helping to release the excessive inhibition of the GPi on the central thalamus [5,63]. The observation that GPe (and not GPi) lesions affect sleep-wake behavior in animal models [8] suggests that the perceived arousal effects of zolpidem in DOC patients are more likely to be due to the activity of the GPe than that of the GPi.

Zolpidem’s impact on the GABA-A receptor rich GP may, instead, have multifaceted downstream effects given the different connectivity profiles of the GPe and GPi. Our previous diffusion tractography work uncovered dissociable patterns of frontal and thalamic connectivity across the GPe and GPi in humans [15]. While the GPe showed robust connections with the prefrontal cortex (PFC) and central thalamic nuclei (many of which project to the PFC), the GPi selectively connected with more motor-related frontal (e.g., motor association cortices) and thalamic structures [15]; this finding has been corroborated by neurophysiological, pathophysiological, and histological studies in animals [13,20,36]. Furthermore, GPe lesions in humans result in reduced metabolism in the prefrontal regions [64] and zolpidem-responsive DOC patients show post-administration changes in prefrontal activity [65,66,67,68]. This evidence suggests that zolpidem’s action on the GP could be dissociable as a function of the subregion: action on the GPi may improve more motor-related aspects of behavior, whereas action on the GPe may underlie more cognitive arousal, permitting patients to provide coherent responses. The GPe’s preferred connectivity with the PFC and prefrontal projecting thalamic nuclei, which are not observed with the GPi, strengthens this hypothesis [15]. 

## 5. Conclusions

The goal of this current work has been to provide the reader with a coherent summary of the evidence that positions the GPe as an important, and previously overlooked, node in the regulation of arousal and sleep-wake transitions. Traditionally regarded as a relay structure, emerging evidence suggests that the GPe plays a critical role in the maintenance of physiological and behavioral arousal by regulating the balance of excitation and inhibition. In addition, recent evidence suggests that the GPe and its prefrontal cortical connections might play a key role in the loss and recovery of consciousness in both healthy volunteers undergoing anesthesia and long-term DOC patients. When considered together, these two lines of evidence suggest a possible reappraisal of the Mesocircuit Hypothesis, a modification which could bolster this framework’s explanatory power along two directions. On the one hand, the inclusion of the GPe could better reconcile neuroscientific data with the puzzling wakefulness-promoting properties of zolpidem. This might be better understood in terms of the critical role of adenosine, dopamine, and the GPe PV+ neurons in sleep-wake transitions that provide mechanistic substantiation behind why sleep-wake disturbances seem isolated to the GPe and not the GPi. On the other hand, by highlighting the neuroanatomical and functional dissociation between the GPi, with its more motor and posterior patterns of connectivity, and the GPe, with its more cognitive and anterior pattern of connectivity, we provide an additional leg to the MH framework that can allow for more cohesive accounts of neurophysiology, such as the evolution of EEG sleep signatures along an anterior-posterior gradient. Additionally, it is intriguing (albeit entirely speculative) to consider the possibility that distinguishing a more motor-based section of the MH (mediated by the GPi) and a more cognitive-based section of the model (mediated by the GPe) could help understand the phenomena in which the cognitive capabilities of a patient are not matched by their behavioral repertoire (e.g., Cognitive Motor Dissociation). Further experimental studies are needed to verify the different hypothesized roles of the GPe circuits in sleep-wake regulation. 

## Data Availability

Not applicable.
